# Method for Assessing Numbness and Discomfort in Cyclists’ Hands

**DOI:** 10.3390/s25154708

**Published:** 2025-07-30

**Authors:** Flavia Marrone, Nicole Sanna, Giacomo Zanoni, Neil J. Mansfield, Marco Tarabini

**Affiliations:** 1 Department of Mechanical Engineering, Politecnico di Milano, 20156 Milano, Italy; nicole.sanna@polimi.it (N.S.);; 2 Department of Engineering, Nottingham Trent University, Nottingham NG11 8NS, UK

**Keywords:** numbness, discomfort, cyclist palsy, cycling, handlebar, hand-transmitted vibration (HTV), vibrotactile perception threshold (VPT), temporary threshold shift (TTS)

## Abstract

Road irregularities generate vibrations that are transmitted to cyclists’ hands. This paper describes a purpose-designed laboratory setup and data processing method to assess vibration-induced numbness and discomfort. The rear wheel of a road bike was coupled with a smart trainer for indoor cycling, while the front wheel was supported by a vibrating platform to simulate road–bike interaction. The vibrotactile perception threshold (VPT) is measured in the fingers, and a questionnaire was used to assess the discomfort in different parts of the hand using a unipolar scale. To validate the method, ten male volunteers underwent two one-hour cycling sessions, one for each of the two handlebar designs tested. VPT was measured in the index and little fingers of the right hand at 8 and 31.5 Hz before and after each session, while the discomfort questionnaire was completed at the end of each session. The discomfort scores showed a strong inter-subject variability, indicating the necessity to combine them with the objective measurements of the VPT, which is shown to be sensitive in identifying the perception shift due to vibration exposure and the differences between the fingers. This study demonstrates the effectiveness of the proposed method for assessing hand numbness and discomfort in cyclists.

## 1. Introduction

Vibration generated by road irregularities is transmitted to cyclists through the handlebar, the seat, and the pedals, contributing to perceived discomfort [1,2]. Discomfort in the hands depends on many factors such as high pressure on the handlebars, insufficient padding, improper handlebar and component positioning, and poor bike fit, including incorrect saddle tilt. These conditions can further lead to “cyclist’s palsy”, a hand neuropathy caused by excessive nerve compression, characterized by symptoms such as transient numbness and weakness in the hands [3,4,5].

Manufacturers are interested in the evaluation of discomfort and numbness to optimize handlebar design, grip viscoelastic characteristics, or to compare the performance of gloves; however, there are no standardized procedures for the measurement, evaluation, and assessment of hand-related discomfort. Research groups have tested and proposed different approaches to characterize the level of transmitted vibration at the bicycle handlebar. A preliminary modal analysis of the bike handlebars in laboratory settings can be useful to ensure the absence of resonance in the frequency ranges where the hand sensitivity to vibration is higher [6], but it does not provide any qualitative information on the possible vibration-related discomfort.

Another approach to evaluate the bike-rider experience is the instrumentation of the bike components in real conditions with sensors at the interface with the cyclist to measure the vibration acceleration and/or forces and eventually compute the transmissibility or the absorbed power and energy [7,8]. However, the lack of repeatability between tests performed in nominally identical conditions prevents the comparison of the effectiveness of different solutions aimed at maximizing the rider’s comfort.

The effect of vibration has also been evaluated with subjective questionnaires [9,10]; scores are generally assigned to the perceived discomfort through numerical/verbal scales. With the same aim, other studies use the alternative forced choice method, which is a discrimination methodology widely applied to estimate the psychometric function of individuals to detect stimulus differences [1,11,12]. Nonetheless, peripheral neurosensory function is often uncorrelated with the perceived discomfort, thus limiting the applicability of questionnaires [13,14].

The vibrotactile perception threshold (VPT) test has been widely used to measure the vibration-induced neurosensorial response [15,16]. This measure is mediated by the presence of various skin mechanoreceptors that are activated depending on the frequency range of the stimulating vibration [17,18]. Their response can be altered temporarily following hand vibration exposure [17,19,20,21] or permanently in the presence of neuropathies [22,23]. To our knowledge, only one study used a clinical method to assess the effect of hand-transmitted vibration (HTV) in mountain bike cycling [24]. After an outdoor session, the authors examined the nerve function using quantitative sensory testing (QST) (i.e., thermal and vibrotactile perception evaluation and grip and pinch test). However, as acknowledged by the authors, there was a limitation due to a logistics delay between the end of the session and the measurement, resulting in the recovery of the vibration effect.

In this study, we introduce a method for evaluating vibration-induced numbness and discomfort in cyclists’ hands under simulated cycling conditions within a controlled laboratory environment. Unlike traditional approaches, this method requires the cyclist to ride the bike on virtual trainers, thereby creating a realistic simulation of the cycling experience. The need for multiple, real-time neurosensorial response measurements leads to the necessity of assessing VPT during the indoor cycling session, along with the evaluation of discomfort through a subjective questionnaire.

## 2. Methods

### 2.1. Design of the Method

To evaluate vibration-induced responses in the cyclist’s hand under controlled laboratory conditions, a purpose-built experimental setup is designed. A prolonged cycling simulation along with vibrational input is necessary to replicate the real conditions experienced by cyclists. For the assessment of numbness and discomfort, a VPT device is used to measure pre- and post-vibration exposure, and a discomfort questionnaire is administered.

Although a 3D moving platform can be used to generate vibrations, a 2D shaker may be sufficient to create a realistic vibration environment, since the vibration is predominantly confined to the bicycle plane [6,8]. The platform would need to support the front wheel of the bike and have a payload capacity larger than 1 kN to support the rider, part of the bike, and to create vibration stimuli compatible with the desired cycling environment. The rear wheel shall be connected to a passive trainer or a smart trainer to reproduce realistic cycling efforts. To guarantee an optimal vibration transmission, the tires’ pressure needs to be controlled and set according to the manufacturer’s specifications.

**Figure 1:**
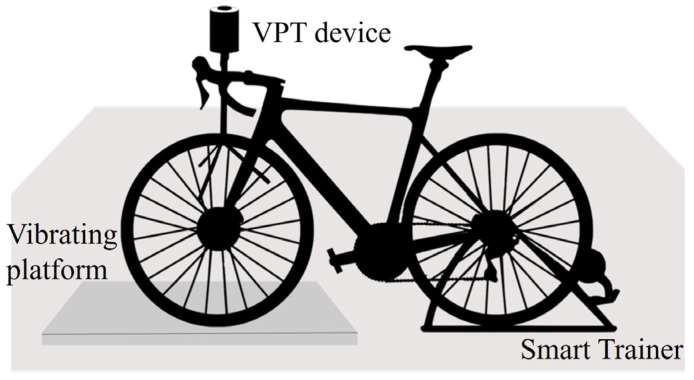
Schematic of the experimental setup.

The areas around the platform must allow the VPT device to be positioned next to the cyclist to administer the test immediately after vibration exposure, without requiring the rider to dismount. The VPT at 8 and 31.5 Hz, the frequencies typically tested [25] within the range of interest, is measured before and after the cycling session in the index and little fingers, which have two different nerve endings and are typically affected during cycling [26,27]. A scheme of the setup is represented in Figure 1.

### 2.2. Questionnaire

Several questionnaires have been used in the literature for the evaluation of comfort. A scoring unipolar scale ranging from absence to strong discomfort can be used to quantify discomfort perceived in the anatomical parts of the hands [28]. To avoid misunderstandings between the numerical value and the adjectival judgment [29], graphical methods can be used. Other comfort evaluation questionnaires used various scoring systems based on a point scale (of 4, 6, or 10 points), such as the Borg scale, or the CP-50 category partitioning scale, sometimes coupled with body maps showing the body regions to be scored [30].

### 2.3. Test Protocol

The protocol consists of cycling sessions in a laboratory environment. Since temperature influences discomfort perception [31], the room temperature has to be recorded before the tests and kept constant and equal among the sessions in order to standardize the experimental conditions. A proper ventilation system is also desirable to provide realistic cooling.

Subjects are required to wear appropriate sports clothing, adjust the setup to their specific characteristics (saddle height, VPT device positioning, etc.) with guidance provided for correct fitting. General participant information (e.g., age, training level, body mass) must be reported.

Before the beginning of the tests, the subject should be familiarized with how to perform the VPT test; skin temperature (T) and VPT in the fingers must be measured in baseline conditions. A cycling session of duration comparable to the outdoor session (minimum 30 min) can be performed at this point. To increase realism, longer sessions in which the subject changes the arm positions with modulated physical effort are desirable.

**Figure 2:**
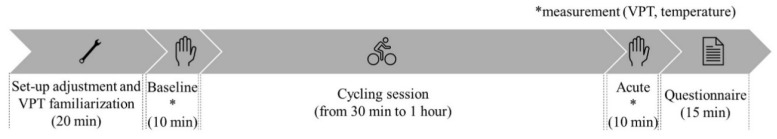
Temporal scheme of the experimental protocol (composed using resources from Canva).

Immediately after the end of the session, temperature and the VPTs in the fingers have to be measured again as acute values and, finally, the subject can answer the discomfort questionnaire. Figure 2 reports the temporal scheme of the experimental protocol.

To prevent the neurosensory response results from being influenced by test adaptation and fatigue, it is recommended that the order of fingers and frequencies tested be randomized among subjects, as well as the order of sessions if each subject has to test different handlebars in different sessions.

### 2.4. Data Processing

To analyze the neurosensorial response, the temporary threshold shift (TTS) of the vibrotactile perception and the variation in skin temperature are computed as follows:

TTS = VPT_Acute_ − VPT_Baseline_ (1)

ΔT = T_Acute_ − T_Baseline_ (2)

The results undergo a statistical analysis of variance and eventual correction (i.e., Tukey test) in order to verify the effects on VPT, TTS, and T of different factors such as the measurement (i.e., baseline and acute), finger (i.e., index and little finger), and test frequency (i.e., 8 and 31.5 Hz). In the case of comparative testing between different handlebars, the handlebar type is considered a further factor.

As with other measures, the variance in questionnaire scores can be analyzed by considering the anatomical parts of the hand as a factor. Given the subjectivity nature of the data, it is advisable to group the results into categories of discomfort (e.g., low, weak, high, strong) to provide a clearer overview of the distribution within the population. Finally, correlation analyses should be conducted to explore potential relationships between VPT/TTS and reported subjective discomfort.

## 3. Method Validation

**Figure 3:**
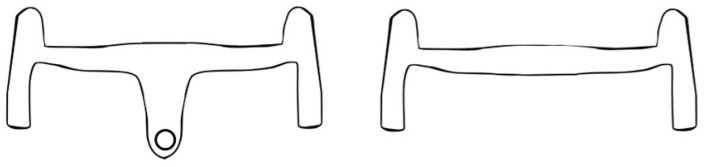
Representation of H-int on the (**left**) and H-stem on the (**right**).

The validation of the method was conducted in the Human Vibration Lab at Polo Territoriale di Lecco-Politecnico di Milano (Lecco, Italy). Tests were conducted to investigate the vibration transmitted to the hand from the bike using two carbon fiber gravel handlebars. The first was an integrated design with the stem and handlebar manufactured as a single component (H-int). The second was a standard design, in which the handlebar and stem were separate components (H-stem) (Figure 3). The handlebars are similar in terms of specific dimensions, with a width of 420 mm, a reach of 120 mm, and a drop of 115 mm. Both handlebars were covered with superlight bar tape and mounted on a road bike installed in the setup previously described.

### 3.1. Experimental Setup

**Figure 4:**
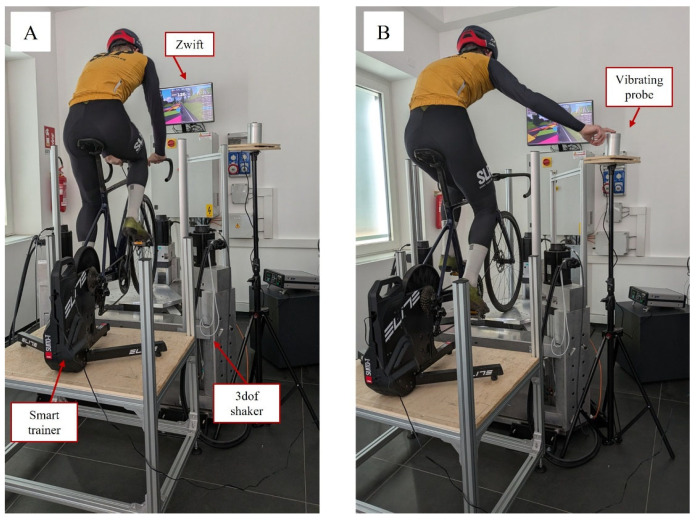
Panel (**A**) depicts the bike mounted on the smart trainer and installed on the shaker, while a subject pedals using the Zwift application on a PC. Panel (**B**) shows the subject performing the VPT test, with the measurement device positioned adjacent to the experimental setup.

The bike was mounted on a smart trainer (Elite Suito-T—Elite-it, Fontaniva, Padua, Italy), which, in turn, was connected to the indoor cycling app Zwift 2024 (Zwift Inc., Long Beach, CA, USA). The trainer was placed on a stable support. The front wheel was mounted on the platform of a 3-dof shaker [32]. Triaxial white noise vibration was generated between 2 and 30 Hz of 0.45, 0.50, and 0.28 m/s^2^ r.m.s. along the anteroposterior, vertical, and mediolateral directions, respectively (Figure 4). The tire pressure was set to 7 bar.

The VPT measurements were performed with the HVLab—Vibrotactile Perception Meter (HVLab, University of Southampton, United Kingdom), which is based on the von Bèkèsy algorithm. We selected a rate of increase and decrease of 3 dB/s (with 5 dB/s for the first reversal), with a maximum duration of each measurement set at 30 s and at least four reversals [16]. The vibrating probe of the HVLab device was positioned on a structure on the side of the bike at a height that allowed the subject to perform the tests while sitting on the bike with the right arm extended horizontally. During the VPT test, the subject was required to apply with the fingertip a constant force on the vibrating probe equal to 2 N and displayed on a scale to ensure consistency.

A discomfort questionnaire (reported in the Appendix A) was designed to quantify the subjective discomfort perceived at the palms, wrists, index fingers, little fingers, and whole hands. The scale consisted of a 10 cm long empty and non-graded bar on which the subject drew a sign corresponding to his/her score, and only then was the corresponding numerical value between 0% = “no discomfort” and 100% = “strong discomfort” assigned employing a proportion. At the end of the survey, there was an open-ended question asking for any further comments about the sensation in the hand.

### 3.2. Experimental Setup

Each subject wore sports clothes and, before starting the tests, got on the bicycle to ensure a proper bike fitting. The height of the saddle and the vibrating probe were adjusted according to the subject’s characteristics: to ensure a consistent posture, saddle height was set to a distance from the bottom bracket obtained by multiplying inseam by 0.883. The saddle’s horizontal position was set by first positioning the cranks so that the pedals were horizontal, with the right pedal at the 3 o’clock position. While the rider was seated on the bike, the ball of the foot was aligned with the pedal axles. The front of the kneecap was then checked for alignment with the pedal axle, using a plumb line. Limitations of the adopted procedures are discussed in Section 5.

**Figure 5:**
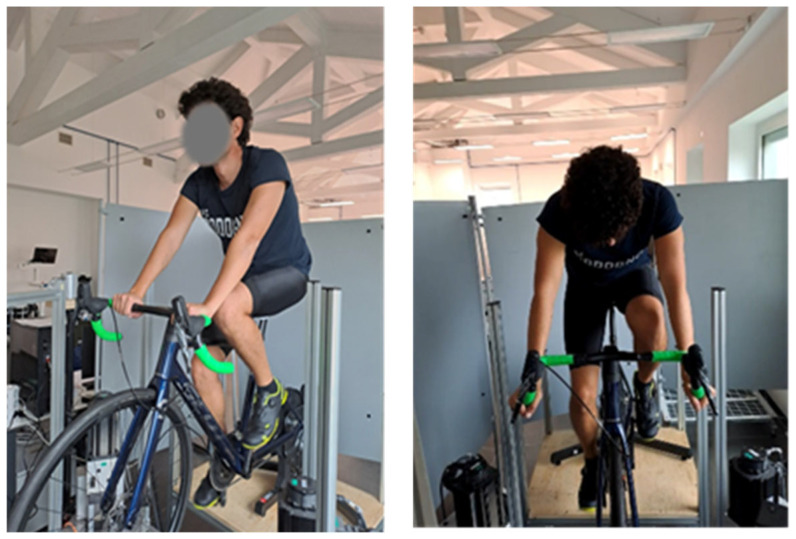
On the (**left**) upper grip and the (**right**) lower grip of the handlebar.

After a period of familiarization with the VPT test, the experiment started by following this protocol:

1. Before the cycling session.
  The skin temperature (T) of the right index and little fingers was acquired using a thermocouple. VPT values at 8 and 31.5 Hz in the index and little fingers were measured in a random order as baseline references. Given the placement of the VPT device on only one side of the bicycle, in proximity to the handlebar, measurements were performed on the right hand for all subjects.
2. Cycling session.
  Participants completed 60 min of pedaling while being exposed to vibration. To control the testing conditions, the smart trainer was used in ERG mode: the trainer adjusted the resisting torque to maintain the target power output independently from the cadence imposed by the cyclist. In detail, there were 5 min of warm-up with increasing power from 85 to 110 W, 50 min at constant power (110 W), and the last 5 min of cool-down decreasing to 85 W. To simulate what happens when riding outdoors, 30 min after the start of the session, the subject was verbally instructed to change the position of the arms for 5 min from the upper (“tops” position) to the lower (“drops” position) grip of the handlebar, as shown in Figure 5. The “tops” and “drops” positions were selected because they allow for the isolation and identification of the specific effects of handlebar materials and geometry on perceived discomfort. Unlike other positions, such as the “hoods”, these eliminate the confounding influence of the brake’s material and structural properties. In “tops” and “drops”, the hands interact exclusively with the handlebar structure itself.
3. After the cycling session.
  Immediately after the end of the session, the fingers’ temperature was measured again and the VPT measurements were repeated as acute values. Finally, the subject answered the discomfort questionnaire.

The entire experiment lasted approximately 3 h, during which both bars were tested. Each subject repeated the protocol twice, once for each handlebar presented in a random order, with a rest period (i.e., sitting on a chair) between them of at least 30 min to ensure complete recovery of the perception shift [19,33], discomfort, and fatigue caused by the previous session.

### 3.3. Participants

The proposed method was tested on ten healthy male subjects who voluntarily participated in the study. To standardize the bike size, participants were selected within a height range of 170 to 180 cm, as recommended by the manufacturer for the chosen frame size. Subjects with musculoskeletal injuries; diseases of the musculoskeletal, cardiovascular, and/or nervous and/or respiratory systems; diabetes; cognitive disabilities or developmental disorders; concussion; vibration-induced diseases; and motion sickness were excluded. The experiments were approved by the Ethics Committee of the Politecnico di Milano (n° 76/2024), and all subjects signed the related informed consent before the start of the tests. Participant demographics (age, weight, and height) were recorded, and their body mass index (BMI) was subsequently computed (Table 1). Table 1 also presents the weekly frequency of general sports exercises performed by the subjects, along with their smoking habits. To be categorized as cyclists, participants were required to own a bicycle and regularly cycle for at least two hours per week, excluding commuting. Furthermore, their knowledge of cycling was assessed, with individuals demonstrating sufficient experience to be categorized as recreational cyclists. Despite the absence of declared pathologies in the informed consent form, the presence of any perception thresholds altered from the sample’s normality at VPT baseline measurement would lead to exclusion from the study. Nevertheless, none of the subjects, not even the cyclists most commonly exposed to vibrations, exhibited such anomalies.

### 3.4. Data Analysis

The TTS at 8 and 31.5 Hz and the ΔT were calculated for the index and the little finger. To evaluate the effect of vibration exposure during pedaling on the objective (i.e., neurosensorial response) and subjective (i.e., questionnaire) data and to verify the applicability of the proposed method with different handlers (i.e., H-int and H-stem), multilevel factorial analysis was completed:

- Effect on VPT: considering the measurement (baseline and acute), finger (index and little finger), and test frequency (8 and 31.5 Hz) as factors. The test was repeated separately for the two handlebars;
- Effect on T: considering the measurement (baseline and acute) and finger (index and little finger) as factors. The test was repeated twice, once for each handlebar;
- Effect on TTS: considering the finger (index and little finger), test frequency (8 and 31.5 Hz), and handlebar (H-int and H-stem) as factors;
- Effect on discomfort scores: considering the anatomical region (palm, wrist, index finger, little finger, and hand) and handlebar (H-int and H-stem) as factors.

In case of significance, the Tukey correction was applied.

Pearson tests were used to correlate the shift in vibrotactile perception and skin temperature and to find a possible relationship between the objective (VPT and TTS at each test frequency) and subjective perception (discomfort score) in the index and little finger. Finally, the effect of the use of tobacco and being a cyclist was evaluated on TTS, ΔT, and discomfort score with one-way ANOVA tests.

All statistical analyses were performed in Minitab^®^ 21.2 (64-bit) with a statistical significance of 0.05.

## 4. Results

**Figure 6:**
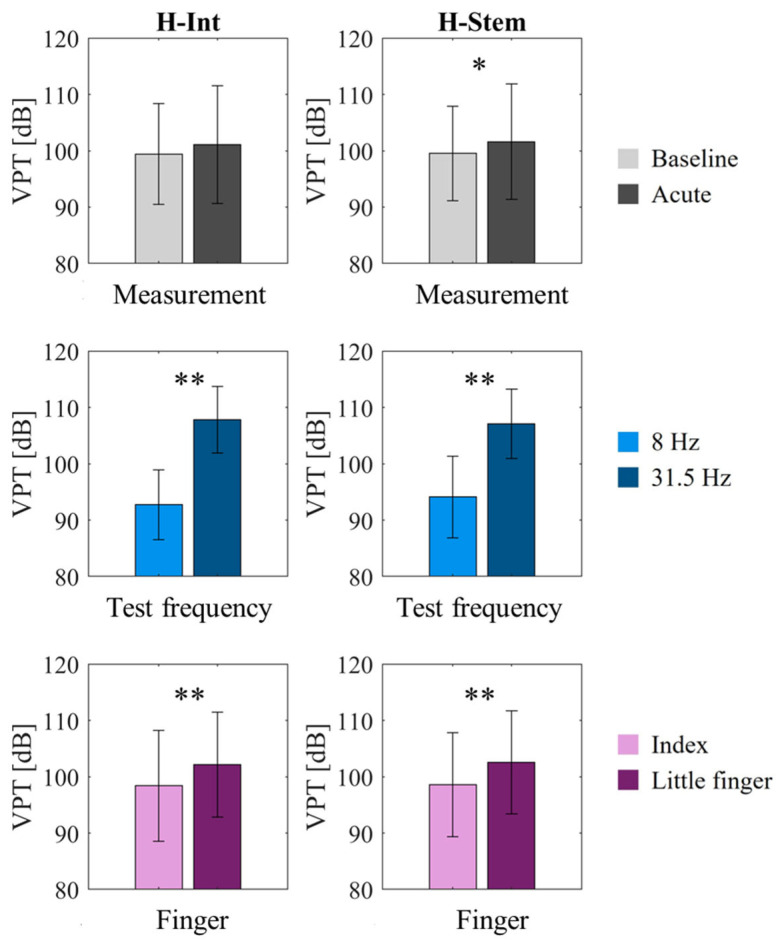
The mean and standard deviation values of VPTs grouped by factors and for each handlebar (H-Int on the left and H-Stem on the right). From top to bottom, aggregated VPT values are grouped by: measurement (i.e., baseline, acute), test frequency (i.e., 8 and 31.5 Hz), and finger (i.e., Index and little finger). In case of significance, * for *p* < 0.05, ** for *p* < 0.01.

The statistical analyses revealed an increase in VPT following a cycling session with vibration exposure (H-int: *p* = 0.052, H-stem: *p* = 0.032). The VPT observed at 31.5 Hz was approximately 14 dB greater than that at 8 Hz and showed a more pronounced effect on the little finger compared to the index finger (*p* < 0.001 for both handlebars). A comparative analysis of the two handlebars for each factor is illustrated in Figure 6.

**Figure 7:**
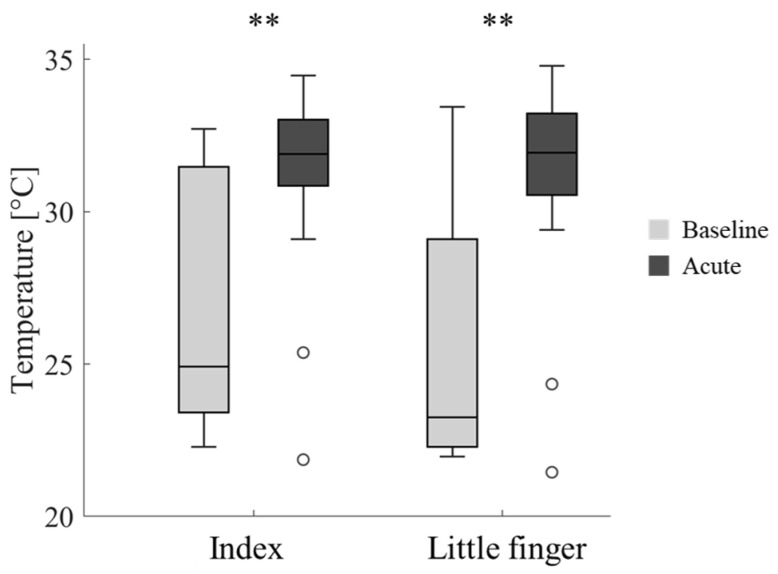
Boxplots of the skin temperature and the outliers recorded at the baseline and acute in the index and little fingers. In case of significance, ** for *p* < 0.01.

Moreover, the skin temperature at the end of the cycling session increased by 4.8 °C (*p* < 0.001) with both handlebars (Figure 7), indicating that the rise in body temperature due to physical exertion predominates over the vasoconstrictive effect of vibration. It should be noted that this observation may not be applicable to other environmental conditions, as colder surroundings could potentially result in hand cooling. No differences between the temperatures of the two fingers were found (H-int: *p* = 0.550, H-stem: *p* = 0.467).

Regarding the effect of vibration exposure on TTS, a significant variation (*p* = 0.008) between 8 Hz (0.5 dB) and 31.5 Hz (3.3 dB) was found; no significant variation was noticed between the two handlebars (*p* = 0.717) and the two tested fingers (*p* = 0.913).

**Figure 8:**
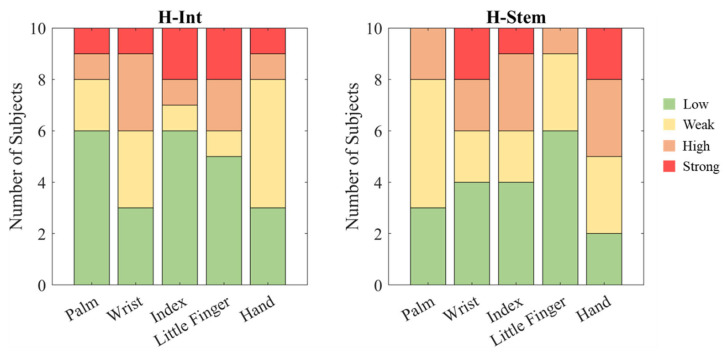
Distribution among the subjects of the scores divided into the intensity classes (0–25% low discomfort, 25–50% weak discomfort, 50–75% high discomfort, 75–100% strong discomfort) for each handlebar.

Opposite results were found in the discomfort scores analysis in which none of the considered factors showed a significant effect (handlebar: *p* = 0.983, anatomical region: *p* = 0.254). Table 2 reported the median and the interquartile range of the discomfort score for each handlebar and anatomical region. Figure 8 shows the distribution among the subjects of the scores divided into four intensity classes (0–25% low discomfort, 25–50% weak discomfort, 50–75% high discomfort, 75–100% strong discomfort).

The open question revealed that six subjects perceived numbness in some or all the fingers, including the thumb which was not considered for the VPT measurements. Four subjects felt discomfort mainly at the wrist and palm of the hands, which, in some cases, resulted improved in correspondence with the change in posture executed in the middle of the test.

No correlation (*p* > 0.05, −0.02 < ρ < 0.25) was found between TTS and ΔT, and between VPT and TTS at each of the test frequencies and discomfort score in the index and little fingers.

The effect of tobacco use was found. Compared to non-smokers, TTS is 3 dB higher (*p* = 0.011), ΔT is 4 °C lower (*p* = 0.002), and the discomfort score is 30.6% higher (*p* = 0.010). Instead, a difference was reported between cyclists and non-cyclists for the ΔT, which was 3 °C higher in the non-cyclist subjects, and for the discomfort score (*p* = 0.033). The cyclists have, on average, a lower discomfort (34.4%) with respect to the non-cyclist participants (43.9%).

## 5. Discussion

We have proposed a method to evaluate vibration-induced discomfort in the hands and fingers with both objective measurements and subjective assessments. The method has been used to compare the characteristics of integrated and traditional road handlebars. Although no correlation between the perceived (discomfort scores) and measured (VPT and TTS) vibration effect was found, both methods showed that vibration exposure during cycling affected the hand.

The VPT measurements were shown to be a sensitive method in discriminating the vibration effects in different fingers before and after cycling. The little finger has higher VPT compared to the index, in discordance with [34], where a correlation between the normative values of the two fingers was found. Also, VPT resulted in being frequency-dependent, with values at 31.5 dB always higher than 8 Hz [35] for both measurement and fingers. VPTs in acute values were higher than in baseline values, with a mean shift of 1.9 dB, demonstrating a worsening of the tactile perception due to vibration exposure. This result is in line with other studies on HTV exposure in the work field [19,36] but in contrast with the study of Fardelin and colleagues [24], who measured the acute effects of mountain bike cycling on hand nervous function with the QST. They found no significant differences between baseline and acute data that could be attributed to a 10–15 min delay between the end of exposure and the execution of the QST due to in-field logistic reasons that were eliminated in the controlled laboratory setup.

The comparison between cyclist and non-cyclist participants showed less ΔT in the latter group. A possible explanation may be attributable to cycling fitness. Specifically, the lower ΔT in cyclists may be due to an imposed workload that is small in comparison to the typical cyclist’s functional threshold power (FTP). Consequently, it could be interesting to test cyclists with different levels of training to examine whether a prolonged vibration exposure during life is associated with different neurosensorial impairments.

### 5.1. Questionnaire

The median score of the questionnaires was less than 50% (low/weak discomfort) for all the anatomical regions of the hand for each handlebar, but due to the subjectivity of the method, the interquartile range showed a strong heterogeneity among the subjects, also in terms of the most affected area. These findings may also be due to the fact that the hand force and posture were not controlled during the test. The coupling force (i.e., the sum of grip and push forces) affects the absorbed energy. In the frequency range tested, the absorption increases with the force and with the diameter of the handle [37], influencing the mechanoreceptors’ response and perceived discomfort. Still, as indicated by the open-ended responses to the questionnaire, a change in posture can reduce the sensation of discomfort. It is known that the hand pressure and load maintained for long periods can cause damage to the ulnar nerve, suggesting that the use of padding gloves and changing the position of the wrist reduce the risk of cyclist’s palsy [38]. Given that numbness occurs as a combined effect of posture and HTV exposure, it would be fundamental in the future to monitor the force at the hands–handlebar interface and the hand/wrist position during the test to characterize specific effects (such as handlebar geometry and type of vibration applied) on discomfort.

Finally, the comparison between cyclist and non-cyclist participants showed greater perceived discomfort in the latter group. The use of tobacco seemed to play a role in a worsening of the vibrotactile perception and skin temperature shift and subjective discomfort, but given the limited number of subjects, the results are not generalizable and need further investigation.

### 5.2. Limitations

Further studies are necessary to refine the proposed approach by considering additional test conditions. Vibration signals could be acquired from the field and later replicated under controlled laboratory conditions. This would allow for the standardization of test conditions that simulate various types of bike–terrain interactions, so that various bicycle models can be tested. In the case of comparative studies in which different ranges of vibration levels and frequencies are applied, the measurements of the vibration at the handlebar can also be performed to correlate the applied vibration levels with the recorded perception and discomfort. In addition, when applying wider frequency ranges during cycling, the measurement of VPT at test frequencies greater than 31.5 Hz could be considered to assess the neurosensory response induced in various mechanoreceptor types [18].

The present study serves as a method validation and did not involve a large and diverse population: future studies should include females and individuals with different anthropometric and demographic characteristics to further validate its applicability. In addition, a better stratification of the population by cycling experience level could be beneficial to verify if negative effects in the medium to long term can be present in professional bikers.

The presence of factors that can influence perception and discomfort, such as posture, hand positioning and force [39,40], would require an optimization of experimental variables control. Considering that force and posture can also influence the contact area between the fingers and the vibrating surface of the handlebar, thereby differently stimulating the skin mechanoreceptor [41], monitoring these aspects is necessary for a better comparison of results. More consistent testing procedures should be implemented, including monitoring of posture and hand force with a motion capture system and force transducers. This could also help to characterize any potential influence of consolidated cycling experience on discomfort confounding factors, such as posture and grip force.

Given that the change in posture was found to be influential in the discomfort evaluation, positions other than “tops” and “drops” have to be considered in the future to better mimic the real-world situation, such as the “hoods”, “hooks”, and “ramps” positions.

Environmental factors may play a significant role in the perception of discomfort. For instance, lower ambient temperatures could exacerbate numbness, while ventilation and glove usage may also influence results. However, considering the obtained results and the fact that the discomfort is more perceived at low frequencies [42] and that the VPT increases with increasing frequency [43], we can conclude that the combined use of the two methods may be promising for a comprehensive assessment of the effect of vibration on the cyclist’s hands.

## 6. Conclusions

This work proposed a method to investigate numbness and discomfort in the cyclist’s hands, using a subjective questionnaire and VPT measurements in the fingers, by simulating cycling sessions in a controlled laboratory environment. A validation study was performed to check the effectiveness of the method. Although further studies under different test conditions are required, the proposed approach provides a viable method for evaluating the impact of different bicycle components on cyclists’ perceived hand discomfort. Consequently, this methodology may be valuable for manufacturers in optimizing bicycle components and accessories, such as handlebars, gloves, and handlebar tapes.

## Figures and Tables

**Table 1:** Anthropometric and physical activity characteristics and smoking habits for each participant. Bold is used to indicate the participants who are cyclists.

Subject ID	Age [Years]	Body Mass [kg]	Height [cm]	BMI [kg/m^2^]	Training [Hours/Week]	Smoke Habits [Cigarettes/Day]
1	24	72	173	24.1	5	7
**2**	**26**	**75**	**173**	**25.1**	**3.5**	**-**
3	24	76	180	23.5	2	-
4	26	76	177	24.3	4	4
**5**	**25**	**72**	**176**	**23.2**	**6**	**2**
**6**	**22**	**76**	**180**	**23.5**	**3**	**-**
7	22	78	180	24.1	5	-
8	23	73	173	24.4	3	-
9	25	70	176	22.6	4	-
**10**	**50**	**76**	**176**	**24.5**	**3**	**-**
Total Mean ± SD	26.7 ± 8.3	74.4 ± 2.5	176.4 ± 2.9	23.9 ± 0.7	3.9 ± 1.2	-

**Table 2:** Median (interquartile range) of the questionnaire score for each anatomical region and handlebar. Each score is expressed in percentage between 0% = “No Discomfort” and 100% = “Strong Discomfort”.

	H-Int [%]	H-Stem [%]	Total [%]
Palm	21.7 (12.6–41.8)	33.0 (18.9–44.9)	28.3 (14.1–45.4)
Wrist	33.3 (20.1–67.6)	47.3 (7.9–69.2)	39.0 (9.4–69.6)
Index finger	20.2 (6.0–63.7)	46.4 (10.4–88.4)	27.4 (8.6–77.4)
Little finger	23.5 (6.3–66.1)	19.0 (8.6–42.6)	19.0 (7.4–50.0)
Whole hand	39.9 (20.1–49.7)	55.1 (34.2–79.8)	42.3 (27.1–68.6)

## Data Availability

Dataset available on request from authors.

## Abbreviations

The following abbreviations are used in this manuscript:

BMI	body mass index
FTP	functional threshold power
HTV	hand-transmitted vibration
QST	quantitative sensory testing
TTS	temporary threshold shift
VPT	vibrotactile perception threshold
